# Blood-Based DNA Methylation Analysis by Multiplexed OBBPA-ddPCR to Verify Indications for Prostate Biopsies in Suspected Prostate Cancer Patients

**DOI:** 10.3390/cancers16071324

**Published:** 2024-03-28

**Authors:** Markus Friedemann, Carsten Jandeck, Lars Tautz, Katharina Gutewort, Lisa von Rein, Olga Sukocheva, Susanne Fuessel, Mario Menschikowski

**Affiliations:** 1 Institute of Clinical Chemistry and Laboratory Medicine, Medical Faculty Carl Gustav Carus, TUD Dresden University of Technology, Fetscherstr. 74, 01307 Dresden, Germany; carsten.jandeck@uniklinikum-dresden.de (C.J.); katharina.gutewort@mailbox.tu-dresden.de (K.G.); l.rein@blutspende.de (L.v.R.); 2 Joint Practice of Urology “Am Blauen Wunder”, Schillerplatz 2, 01309 Dresden, Germany; 3 Department of Hepatology, Royal Adelaide Hospital, Port Rd., Adelaide, SA 5000, Australia; olga.sukocheva@sa.gov.au; 4 Clinic of Urology, Carl Gustav Carus University Hospital, TUD Dresden University of Technology, Fetscherstr. 74, 01307 Dresden, Germany; susanne.fuessel@uniklinikum-dresden.de

**Keywords:** liquid biopsy, prostate cancer, circulating cell-free DNA (cfDNA), DNA methylation, cell-free tumour DNA, digital PCR

## Abstract

**Simple Summary:**

A multiplexed optimised bias-based preamplification–digital droplet PCR (OBBPA-ddPCR) diagnostic technique was developed and tested in this study. The method estimates blood-derived cell-free DNA (cfDNA) methylation levels of *RASSF1A*, *MIR129-2*, *NRIP3*, and *SOX8* target sequences in blood samples. The study included 90 healthy individuals, 40 benign prostatic hyperplasia patients, and 39 prostate cancer (PCa) patients. The risk scores were developed to further reduce unnecessary prostate biopsies and PCa overdiagnosis. The scores included classical risk factors and methylation data, delivering an improved 70% specificity for BPH patients and 100% sensitivity for clinically significant PCa patients.

**Abstract:**

Current prostate carcinoma (PCa) biomarkers, including total prostate-specific antigen (tPSA), have unsatisfactory diagnostic sensitivity and specificity resulting in overdiagnosis and overtreatment. Previously, we described an optimised bias-based preamplification–digital droplet PCR (OBBPA-ddPCR) technique, which detects tumour DNA in blood-derived cell-free DNA (cfDNA) of cancer patients. The current study investigated the performance of newly developed OBBPA-ddPCR-based biomarkers. Blood plasma samples from healthy individuals (*n* = 90, controls) and PCa (*n* = 39) and benign prostatic hyperplasia patients (BPH, *n* = 40) were analysed. PCa and BPH patients had tPSA values within a diagnostic grey area of 2–15 ng/mL, for whom further diagnostic validation is most crucial. Methylation levels of biomarkers *RASSF1A*, *MIR129-2*, *NRIP3*, and *SOX8* were found significantly increased in PCa patients compared to controls. By combining classical PCa risk factors (percentage of free PSA compared to tPSA (QfPSA) and patient’s age) with cfDNA-based biomarkers, we developed PCa risk scores with improved sensitivity and specificity compared to established tPSA and QfPSA single-marker analyses. The diagnostic specificity was increased to 70% with 100% sensitivity for clinically significant PCa patients. Thus, prostate biopsies could be avoided for 28 out of 40 BPH patients. In conclusion, the newly developed risk scores may help to confirm the clinical decision and prevent unnecessary prostate biopsy.

## 1. Introduction

The incidence of prostate carcinoma (PCa) is 14.1%, the second highest incidence of cancer in men worldwide, which converts into 1.4 million new cases per year. The PCa mortality rate is 6.8% (375,000 deaths per year), making PCa the fifth leading cause of cancer-related death worldwide [1]. Aside from the established mutations in BRCA1 and BRCA2 genes, PCa risk factors are limited and general in nature, including patient’s age, ancestry, and lifestyle factors [2]. Currently used PCa markers often demonstrate unsatisfactory diagnostic sensitivity (SEN) and specificity (SPE), indicating their suboptimal diagnostic capacity for disease detection and stratification. The level of total serum prostate specific antigen (tPSA) is the established parameter for the early diagnosis of PCa, which has a high SEN of 93% but a low SPE of only 20% (4 ng/mL as cutoff) [3]. Numerous factors can cause an unspecific tPSA increase, such as patient’s age, individual prostate volume, acute and/or chronic prostatitis, benign prostatic hyperplasia (BPH), and mechanical prostate manipulations [4]. Accordingly, recent studies have shown that tPSA-based overdiagnosis resulted in 65–70% unnecessary prostate biopsies [5,6,7,8]. Almost 13% of the assessed men had false-positive PCa diagnosis after three to four rounds of tPSA-based screening [9], highlighting the limitation of this diagnostic method.

It is essential to limit the number of unnecessary prostate biopsies as 0.5–1.0% of these procedures are accompanied by clinically significant infections, bleeding, and/or urinary retention [9]. The other limitation of this method is associated with false-negative diagnostics. It has been estimated that 15% of men with a normal serum tPSA level below 4 ng/mL and a cancer-negative result on digital rectal examination had biopsy-proven PCa [10]. Furthermore, no significant difference in PCa mortality 10 years after initial diagnosis was associated with tPSA screening compared to controls not undergoing a PSA screening, although the detection of PCa cases at low aggressive risk of progression was increased [11]. The data suggest that current tPSA-based screening practices cannot reliably distinguish between indolent disease and life-threatening PCa. Even though imaging methods, like multiparametric magnetic resonance imaging, can improve SPE to 74% with 88% SEN [12], there is still an urgent need for new biomarkers with improved performance to predict clinical outcomes of PCa disease.

Various epigenetic changes occur during early carcinogenesis, leading to increased survival and progression of cancer cells. Analysis of aberrant DNA methylation patterns has high diagnostic potential as a reliable biomarker for the onset of carcinogenesis and disease progression [13,14,15,16,17,18]. Previously, we described a new technique, named OBBPA-ddPCR, consisting of an optimised bias-based preamplification (OBBPA) step followed by digital droplet PCR (ddPCR) to detect minute amounts of aberrantly methylated tumour DNA fragments against a high background of wild-type DNA in the blood-derived circulating cell-free DNA (cfDNA) of cancer patients [19,20]. The OBBPA-ddPCR technique indicated a superior sensitivity in early detection of PCa metastases [19]. The aim of the current study was to investigate the performance of a newly developed OBBPA-ddPCR-based biomarker panel, consisting of five different genomic regions. The current study analysed blood plasma samples from healthy individuals as well as PCa and BPH patients with tPSA values ranging from 2 to 15 ng/mL, for whom further diagnostic validation is most crucial [21,22].

## 2. Materials and Methods

### 2.1. Blood Plasma Sample Collection and Analysis

For epigenetic biomarker selection, Illumina Infinium MethylationEPIC array analyses were conducted in cooperation with the group of Prof. D. Capper (Charité—Universitätsmedizin Berlin, Department of Neuropathology). We analysed 5 blood serum pools from healthy male individuals (taken during medical training for blood collection) aged ≤30 years to reduce the risk of undetected cancer. Furthermore, 6 blood serum pools from patients with advanced PCa aged between 57 and 78 years were analysed. Each pool comprised a total of 2 mL blood serum and included samples from two individuals. The average tPSA values in PCa serum pools were 7.9, 25.5, 85.7, 636.9, 730, and >1000 ng/mL, to ensure sufficient amounts of aberrantly methylated cfDNA fragments. Following the extraction (see Section 2.4), 200 ng of isolated cfDNA was examined for each measurement using a standard Illumina protocol.

For the evaluation of the newly identified biomarker sequences, we initially collected both serum and EDTA plasma samples from the patients for this prospective study. Since we found significantly higher levels of tumour cfDNA copies and lower amounts of background cfDNA in the plasma samples compared to the serum samples in the first investigations (unpublished data), we subsequently included only EDTA plasma samples in the analyses of this study. Using the standard procedure, 2–5 mL of blood plasma from 132 suspected PCa patients of a urological practice (“Am Blauen Wunder Dresden”, Dresden, Saxony, Germany) and 90 healthy individuals (taken during medical training for blood collection from medical students ≤30 years of age and without any preexisting urological diseases) was collected and stored at 80 °C until cfDNA isolation. It has been found that 124 out of 132 patients had serum tPSA values in the range of 2 to 15 ng/mL at the time of blood sampling. These patient samples were further subdivided into two groups, including BPH (*n* = 40) and PCa (*n* = 39), following routine histopathological examination. The PCa group was further subdivided into indolent (*n* = 7) and clinically significant PCa (*n* = 32) groups. Indolent PCa patients received no PCa-related treatment (“active surveillance”) and were defined to have a Gleason score (GS) of 6, tPSA values <10 ng/mL, and T1/T2a cancer stage. Notably, 45 patients received no histopathological examination up to the time of data evaluation for the present study (September 2023) and were treated as an unknown control group. Additionally, 8 patients diagnosed with clinically significant PCa and with tPSA values ≥15 ng/mL were included as a positive control group.

Clinical characteristics are summarised in Table S1. The analysis was approved by the Ethics Committee of the Technische Universtiät Dresden (Dresden, Germany).

### 2.2. In Silico Biomarker Selection

To identify differentially methylated DNA regions in liquid biopsies of PCa patients, Level 3 preprocessed TCGA Illumina 450k methylation data from prostate tumours (*n* = 497) were re-analysed (https://www.cancer.gov/tcga (accessed on 11 February 2019)) [23]. The results were compared with TCGA data from normal prostate tissue (*n* = 50) [23]. Since the majority of cfDNA comprises blood cell genomic DNA [24,25], we used data from the GEO dataset GSE87571, which includes white blood cell data from 421 individuals and 732 samples (ranging in age from 14 to 94 years old) to simulate the genomic background methylation of cfDNA [26]. However, all tissues can potentially release DNA into the bloodstream [27]. Therefore, we included methylation data from Level 3 preprocessed TCGA Illumina 450k methylation datasets from normal bladder (*n* = 21), kidney (*n* = 205), liver (*n* = 50), and lung tissues (*n* = 74) [23] in our analysis for biomarker selection criteria. A custom Python script (Python 3.7.3) was used to exclude unwanted CpG sites from the Illumina 450k methylation dataset according to the “MASK.general” option of the GPL13534 manifest file (accessed on 7 March 2019) [28].

### 2.3. Preparation and Usage of External dsDNA Standard

A short synthetic dsDNA sequence (112 bp) and its multimeric ligation products (112 bp to 448 bp length) were used as quality control for cfDNA isolation and bisulphite modification. The ssDNA sequence was synthesised by Eurofins Genomics (Ebersberg, Germany) and was as follows:

5′-GGAGGTTGTGATTGAGGTTATGGGGCAAGGAAGGTGGAGACTCTGCGT-CGCGGAGCAGCTGGGTGTGTTTACCAGAATCCAGGTGGTTTGCATTTGGGTAGGGTAAGTAGGA-3′. The ssDNA was amplified by a standard PCR reaction using CpG-independent forward (5′-GGAGGTTGTGATTGAGGTTA-3′) and reverse primers (5′-TCCTACTTACCCTACCCAAA-3′). The PCR product was isolated with the DNA Clean & Concentrator-5 Kit (Zymo Research; Freiburg, Germany), treated with T4 Polynucleotide Kinase (NEB, Frankfurt am Main, Germany), and ligated using Blunt/TA Ligase Master Mix (NEB, Frankfurt am Main, Germany) to simulate fragment size distribution of cfDNA. A defined amount of isolated standard DNA fragments was spiked into the blood plasma samples and the recovery was measured after bisulphite modification by ddPCR [20] using the aforementioned forward and reverse primers together with a 5′-HEX-modified oligonucleotide probe (5′-HEX-CCCAACTACTCCACAACACAA-BHQ-3′), which was specific for the bisulphite-modified DNA standard sequence.

### 2.4. Isolation of cfDNA from Blood Samples

Isolation of cfDNA from 2 mL blood serum pools or 2–5 mL of individual serum and plasma samples was performed using the NucleoSnap DNA Plasma Kit (Macherey-Nagel, Düren, Germany) according to the manufacturer’s instructions with an elution volume of 40 µL nuclease-free water. All DNA samples were stored at −80 °C until bisulphite modification procedures.

### 2.5. Bisulphite Modification of Isolated cfDNA

DNA concentration was determined using a Quantus photometer and QuantiFluor dsDNA-System Kit (Promega, Mannheim, Germany). Bisulphite modification was conducted using an EpiTect Fast DNA Bisulfite Kit (Qiagen GmbH, Düsseldorf, Germany) according to the manufacturer’s instructions with an elution volume of 40 µL EB buffer. Samples were concentrated and ethanol residues were reduced by incubating the bisulphite-treated DNA for 20 min at 70 °C, reaching a final sample volume of 12 µL. Samples were stored at −80 °C until further analysis.

### 2.6. Preparation of DNA Samples with a 50% Methylation Degree

Standard DNA samples were prepared with equal amounts of unmethylated and methylated control DNA copies (Qiagen GmbH) to quantify the PCR bias as previously described [19,20]. In brief, approximately 50 copies of unmethylated and 50 copies of methylated DNA were utilised during the preamplification step, keeping the copy number below the copy per droplet (CPD) value of 6 in the following ddPCR procedure after 12 cycles of preamplification.

### 2.7. Preamplification of Bisulphite-Modified DNA

Optimisation of preamplification conditions was described previously [19,20]. In brief, the primer annealing temperature was tested between 50 °C to 63 °C using the CFX thermal cycler (Bio-Rad Laboratories GmbH, München, Germany). MgCl_2_ was added to final concentrations of 1.5, 2.5, 3.5, 4.5, and 5.5 mM MgCl_2_. Blood plasma samples from healthy individuals and BPH or PCa patients were analysed using an optimised annealing temperature of 58.3 °C and an optimised MgCl_2_ concentration of 3.5 mM. The total PCR reaction volume was 25 µL, including 0.125 µL of 5 U/µL HotStarTaqPlus (Qiagen GmbH), 200 µM of dNTPs, and 400 nM of forward and reverse primers of all 5 target sequences (Table S2). The thermal cycling conditions were as follows: 95 °C for 5 min and 15 cycles at 94 °C for 10 s; different annealing temperatures as indicated for 30 s, followed by 72 °C for 30 s; and a final hold at 4 °C. Optimisation analyses were repeated three times. Blood plasma samples were analysed as duplicates with 4 µL of concentrated, bisulphite-modified patient cfDNA. In each run, 10 ng bisulphite-modified unmethylated (0%) and methylated (100%) standard DNA (Qiagen GmbH) was used as positive controls together with no template controls. Genomic DNA without bisulphite modifications was used as additional negative controls.

### 2.8. ddPCR of Bisulphite-Modified DNA

All ddPCR analyses were performed using the QX100 Droplet Digital PCR System (Bio-Rad) according to the manufacturer’s instructions and as described previously [19,20]. Probe sequences were designed using OligoArchitect^TM^ Online software (http://www.oligoarchitect.com (accessed on 12 June 2019)) from Sigma-Aldrich (Taufkirchen, Germany) and were synthesised with BHQ-1 as a fluorescence quencher at the 3′-end as well as 5′-FAM for methylated sequences or 5′-HEX modifications for detection of unmethylated sequences (Table S2). The optimal ddPCR annealing temperatures for the 5 target regions were determined in preliminary experiments using 0% and 100% methylated standard DNA controls (Qiagen GmbH) (Table S2). Primer and probes were used at final concentrations of 900 nM and 250 nM, respectively. The thermal cycling conditions included 95 °C (10 min), 40 cycles of 94 °C (30 s), and 50.7 °C (1 min) with a final 10 min hold at 98 °C. All methylation quantification experiments included no template and genomic DNA controls. Data were analysed using QuantaSoft version V1.6.6.0320 (Bio-Rad). To minimise inter- and intra-experimental variability, fluorescence values were calculated relative to the individual, well-specific baseline fluorescence, using a custom Python script (Python 3.7.3). We defined that two separate positive OBBPA-ddPCR measurements per sample with more than 5 positive droplets are required to be considered as a positive signal. The definition was set to avoid overrepresentation of marginal amounts of methylated target DNA due to the stringent PCR bias and subsequent strong amplification of methylated DNA fragments in the preamplification step.

### 2.9. Data Analysis

“Centre values” were defined as means with standard deviations as error indication. Normal distribution was analysed using the Shapiro–Wilk test [29]. Levene’s test was used to assess the equality of variances between different groups. Normally distributed, homoscedastic data were analysed using one-way ANOVA followed by Tukey’s test if significant differences with *p* < 0.05 were detected. Normally distributed, heteroscedastic data were analysed by Welch’s ANOVA followed by a Games–Howell post hoc test when Welch’s ANOVA indicated significant differences with *p* < 0.05. Non-normally distributed, heteroscedastic data were analysed using a Kruskal–Wallis test and Conover’s post hoc test. Levels of significance were defined and indicated as *p* < 0.05 (*), *p* < 0.01 (**), and *p* < 0.001 (***).

Receiver operating characteristic (ROC) curve analyses were conducted and areas under the curve (AUCs) were calculated using the modules “roc_curve” and “auc” from the Scikit-learn library (Python 3.7.3).

The fractional abundance of methylated DNA fragments was defined as the percentage of methylated DNA fragments and the sum of methylated and unmethylated DNA fragments of the respective biomarker. In the current study, we developed two PSA-independent (piRISK1/2) and two PSA-dependent PCa risk scores (PRISK1/2). All newly developed scores included methylated *RASSF1A*, *MIR129-2*, *NRIP3*, and *SOX8* copies/mL, the amount of cfDNA/mL, and patient’s age. In addition, PSA-dependent PRISK1/2 comprised the assessment of the percentage of free PSA (fPSA) compared to tPSA (QfPSA).

## 3. Results

### 3.1. In Silico Biomarker Selection

**Figure 1:**
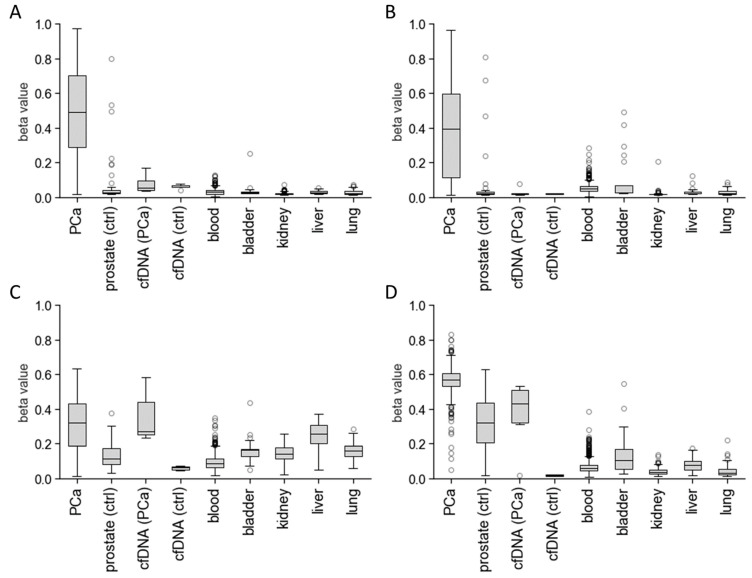
In silico biomarker panel selection. To identify differentially methylated DNA regions in blood liquid biopsies of PCa patients, TCGA Illumina 450k methylation datasets from prostate tumours (PCa, *n* = 497) and normal prostate (prostate (ctrl), *n* = 50), bladder (*n* = 21), kidney (*n* = 205), liver (*n* = 50), and lung tissues (*n* = 74) were re-analysed [23]. The genomic background DNA methylation in liquid biopsies was simulated using Illumina 450k methylation data from the GEO dataset GSE87571, which includes white blood cells from 421 individuals and 732 samples ranging in age from 14 to 94 years old [26]. Additionally, we analysed 5 blood serum pools from healthy male individuals (cfDNA (ctrl)) and 6 blood serum pools from PCa patients (cfDNA (PCa)) using the Illumina Infinium MethylationEPIC array. Methylation beta values from representative CpG sites are presented as box plots for the biomarkers/CpG sites *CCDC181*/cg00002719 (**A**), *MIR129-2*/cg14416371 (**B**), *NRIP3*/cg03963327 (**C**), and *SOX8*/cg08965276 (**D**). Box plots consist of the median (“centre value”), the 25th and 75th percentiles (box edges), and the 10th and 90th percentiles (whisker boundaries). Data points outside whisker boundaries are shown as circles.

The Infinium^®^ HumanMethylation450k BeadChip assay covers over 450,000 DNA methylation sites at single-nucleotide resolution. We re-analysed Illumina 450k methylation datasets derived from The Cancer Genome Atlas (TCGA) [23] for PCa tissues (*n* = 497), compared to healthy prostate tissue samples (*n* = 50) as well as a collection of other healthy tissues (bladder (*n* = 21), kidney (*n* = 205), liver (*n* = 50), and lung tissues (*n* = 74)), which may contribute to the cfDNA methylation pattern (Figure 1).

DNA methylation data from white blood cells of 421 healthy individuals (GSE87571) were included to simulate background DNA methylation of cfDNA [26]. Moreover, Infinium^®^ MethylationEPIC analyses of over 900,000 CpG sites were conducted for five blood serum pools from healthy male individuals and six pools from PCa patients. All selected CpG sites exhibited increased CpG methylation levels for PCa tissues which were significantly different from healthy prostate tissues as well as normal bladder, kidney, liver, and lung tissue data (*p* < 0.001). Only *NRIP3*/cg03963327 showed an elevated CpG methylation of 24.9 ± 7.7% for normal liver tissue with no significant differences compared to PCa methylation levels (31.1 ± 15.3%). Blood serum pools from PCa patients had significantly increased methylation levels compared to serum pools from healthy individuals for *NRIP3*/cg03963327 and *SOX8*/cg08965276 (*p* < 0.001). Moreover, methylation levels of healthy individuals were lower compared to the cancer-patient samples for all selected CpG sites, ranging from 1.8 ± 0.5% to 6.0 ± 1.1% for blood serum-derived cfDNA and 3.1 ± 1.6% to 9.3 ± 4.3% for genomic DNA from white blood cell samples. Low baseline methylation levels in the healthy controls and significant methylation differences between PCa and healthy tissues indicate the reliability of the selected markers.

### 3.2. Analysis of Preamplification PCR Biases Using Primer Design, MgCl_2_ Concentration, and Annealing Temperature Optimisation

**Figure 2:**
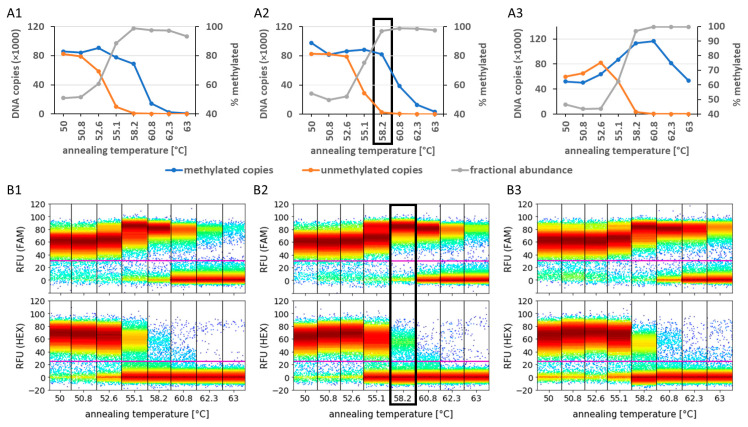
Optimising PCR bias by adjusting MgCl_2_ concentration and annealing temperature. The impact of varying Mg^2+^ concentrations and primer annealing temperatures on the amplification rate of methylated and unmethylated *NRIP3* DNA sequences is shown as an example of multiplex preamplification optimisation. (**A1**–**A3**) DNA fragment numbers of methylated (blue) and unmethylated DNA (orange) as well as the percentage of methylated DNA relative to the total DNA after 12 cycles of preamplification (PCR bias, grey) are displayed as function of varying primer annealing temperatures for Mg^2+^ concentrations of 2.5 mM (**A1**), 3.5 mM (**A2**), and 4.5 mM (**A3**). (**B1**–**B3**) Fluorescence signals of FAM fluorophores for methylated (top) and HEX fluorophores for unmethylated DNA sequences (bottom) are shown depending on the primer annealing temperature and varying Mg^2+^ concentrations of 2.5 mM (**B1**), 3.5 mM (**B2**), and 4.5 mM (**B3**). Optimal preamplification conditions (58.2 °C and 3.5 mM Mg^2+^) are indicated using black frames.

Previously, we reported optimisation details of individual PCR preamplification reactions for *RASSF1A* and *GSTP1* [20]. In the current study, we analysed the impact of varying Mg^2+^ concentrations (1.5–5.5 mM) and annealing temperatures (50–63 °C) on the amplification efficiency of methylated and unmethylated standard DNA and the resulting PCR biases for the multiplex amplification of five biomarkers: *RASSF1A*, *CCDC181*, *MIR129-2*, *NRIP3*, and *SOX8* (Figure 2 and Figure S1). To demonstrate the optimisation process, multiplex preamplification results for *NRIP3* with Mg^2+^ concentrations of 2.5–4.5 mM are shown in Figure 2.

PCR bias was defined as the percentage of methylated fragments relative to the total amount of target DNA fragments after 12 preamplification cycles. The main goal for the optimisation experiments was to find reaction conditions for all 5 marker sequences as follows: (1) the PCR bias is above 90%, (2) methylated DNA sequence amplification efficiencies and corresponding fluorescence signal amplitudes are near the optimum, and (3) significant amounts of unmethylated DNA (≥500 copies after 12 preamplification cycles) with sufficient fluorescence signal amplitudes are detectable as internal controls. The last requirement was defined to avoid false-positive results due to exclusive methylation-specific amplification. Generally, an increased primer annealing temperature and/or a decreased Mg^2+^ concentration led to an increased PCR bias due to increased differences between the PCR amplification efficiencies of methylated and unmethylated DNA sequences in favour of methylated DNA. Considering reaction conditions with PCR biases above 90%, maximum amplification efficiencies of methylated DNA together with optimal fluorescence signal amplitudes were observed from 55.1–58.2 °C and Mg^2+^ concentrations of 3.5–4.5 mM for all five biomarker sequences. Amplification efficiencies of methylated biomarker sequences were slightly higher at 4.5 mM Mg^2+^ (compared to 3.5 mM Mg^2+^) at a primer annealing temperature of 58.2 °C. We selected a primer annealing temperature of 58.2 °C and a Mg^2+^ concentration of 3.5 mM as the most stringent PCR reaction conditions at which significant amounts of unmethylated DNA sequences ≥500 copies were detectable with sufficient fluorescence signal amplitudes after 12 preamplification cycles (Figure S1).

### 3.3. Biomarkers’ Methylation in Healthy Individuals and Patients with BPH or PCa

**Figure 3:**
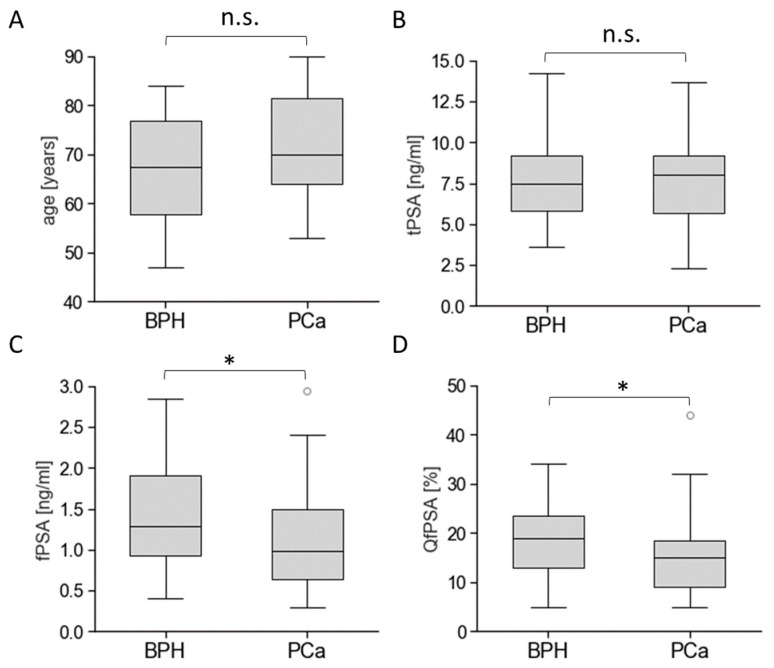
Comparisons of PCa risk factor and routine PCa biomarkers between BHP and PCa groups. Patient’s age (**A**) and tPSA (**B**) were routinely analysed to assess prostate biopsy indication. Additionally, the unbound, free PSA (fPSA) (**C**) and the percentage of fPSA compared to tPSA (QfPSA (**D**)) were determined as secondary biomarkers to further confirm the indication for prostate biopsy. Results are shown for BPH (*n* = 40) and PCa patients (*n* = 39) with tPSA values ranging from 2 to 15 ng/mL. Box plots represent the median (“centre value”), the 25th and 75th percentiles (box edges), and the 10th and 90th percentiles (whisker boundaries). The symbol * indicates significant differences at *p* < 0.05. The abbreviation n.s. (not significant) marks no significant differences found between the analysed cohorts.

We analysed clinical data for 90 healthy individuals, 40 BPH patients, 47 PCa patients, and 45 patients without defined histopathological cancer status. The PCa cohort was further subdivided into groups with tPSA values ranging from 2 to 15 ng/mL (indolent (*n* = 7) and clinically significant PCa patients (*n* = 32)), and eight clinically significant PCa patients with tPSA values ≥15 ng/mL (positive controls). Comparisons of patient age (PCa risk factor) and PSA-based PCa biomarkers between BPH and PCa groups are shown in Figure 3.

A serum tPSA value < 15 ng/mL for BPH and PCa patients was not a sufficient indication for prostate biopsy for the analysed cohorts as we did not observe significant tPSA differences between these cohorts (*p* = 0.849). Patient age tended to be slightly higher in the PCa group (71.8 ± 9.7 years) compared to the BPH group (67.4 ± 10.5 years), with no significance at *p* = 0.057. This observation corresponded the definition of patient’s age as a PCa risk factor [30]. Furthermore, fPSA and QfPSA were significantly decreased (*p* < 0.05) in the PCa cohort compared to those in BPH patients.

**Figure 4:**
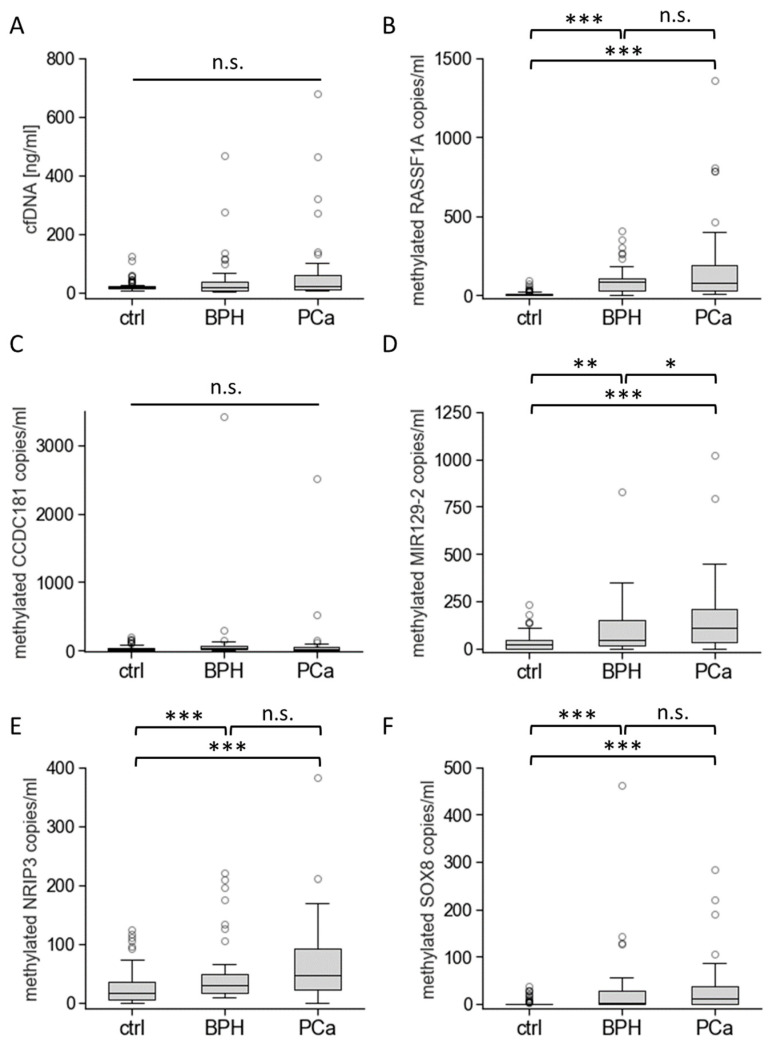
Analyses of total cfDNA amounts and methylated DNA biomarker copies. The total amount of cfDNA/mL (**A**) and the numbers of methylated *RASSF1A* (**B**), *CCDC181* (**C**), *MIR129-2* (**D**), *NRIP3* (**E**), and *SOX8* (**F**) DNA fragments/mL are shown for healthy individuals (ctrl, *n* = 90), BPH (*n* = 40), and PCa patients (*n* = 39) with tPSA values ranging from 2 to 15 ng/mL. Box plots consist of the median as “centre value”, the 25th and 75th percentiles as box edges, and the 10th and 90th percentiles as whisker boundaries. Data points outside whisker boundaries are shown as circles. The symbol * indicates significant differences with *p* < 0.05 (*), *p* < 0.01 (**), or *p* < 0.001 (***). The abbreviation n.s. (not significant) marks no significant differences between the analysed cohorts.

Next, we analysed the amount of cfDNA/mL and the numbers of methylated DNA fragments/mL for the newly defined biomarkers (Figure 4).

To optimise the threshold for the epigenetic biomarkers, we included healthy male individuals in the healthy control group, who were 30 years old or younger at the time of blood sampling. The selection allowed us to reduce the risk of undetected malignant diseases, which increases with a patient’s age. Two additional biomarker sequences, HES5 and EYA4, were excluded from further experiments due to elevated DNA methylation levels in the control group, although in silico analyses had promising results. The average amount of cfDNA/mL increased in the control (22.0 ± 17.7 ng/mL), BPH (47.4 ± 84.0 ng/mL), and PCa groups (75.0 ± 135.6 ng/mL) but no significant differences were detectable. All analysed cfDNA-based biomarkers exhibited gradually increased levels of methylation for the control, BPH, and PCa groups, except methylated *CCDC181*, indicating slightly (but not significantly) increased methylation levels in BPH patients compared to the control and PCa groups. Significant differences (*p* < 0.001) were observed for methylated *RASSF1A*, *NRIP3*, and *SOX8* DNA sequences in BPH and PCa patients compared to those in the control group. Significant differences between BPH (*p* < 0.01) or PCa (*p* < 0.001) and the control group were also detected for methylated *MIR129-2*. Moreover, methylated *MIR129-2* copies were significantly increased (*p* < 0.05) in the PCa cohort (172.0 ± 206.4 copies/mL) compared to the BPH group (107.7 ± 151.1 copies/mL).

Following this, ROC curve analyses were conducted. SEN, SPE, and AUC were utilised to compare the performance of classical PCa biomarker QfPSA with the newly developed cfDNA-based biomarker panels. *CCDC181* was excluded from further analyses because the average DNA methylation for this marker tended to be increased in the BPH group (128.6 ± 529.9 copies/mL) compared to the PCa group (108.5 ± 399.7 copies/mL), thus failing to discriminate between BPH and PCa patients. Single-marker analysis of both classical and cfDNA-based biomarkers produced an insufficient SPE when thresholds were selected to detect all clinically significant PCa patients (100% SEN). Consequently, the DNA methylation biomarkers *RASSF1A*, *MIR129-2*, *NRIP3*, and *SOX8* were combined with the amount of cfDNA/mL and patient’s age to develop two PSA-independent PCa risk scores. PCa risk scores and cutoff values for the individual biomarkers are listed in Table S3. The risk score piRISK1 resulted in 100% SEN for all clinically significant and indolent PCa patients. The risk score piRISK2 offered 100% SEN only for clinically significant but not indolent PCa patients (Figure S2). Significant differences were detected between clinically significant PCa compared to BPH patients for QfPSA (*p* < 0.01) and PCa risk scores piRISK1 and 2 (*p* < 0.001). ROC curve analysis revealed an AUC value of 0.72 when comparing QfPSA values of BPH and indolent PCa patients to the clinically significant PCa cohort. AUC values of PSA-independent scores piRISK1 and piRISK2 were increased compared to QfPSA measurement alone with AUC values of 0.75 and 0.77, respectively (Figure S2).

**Figure 5:**
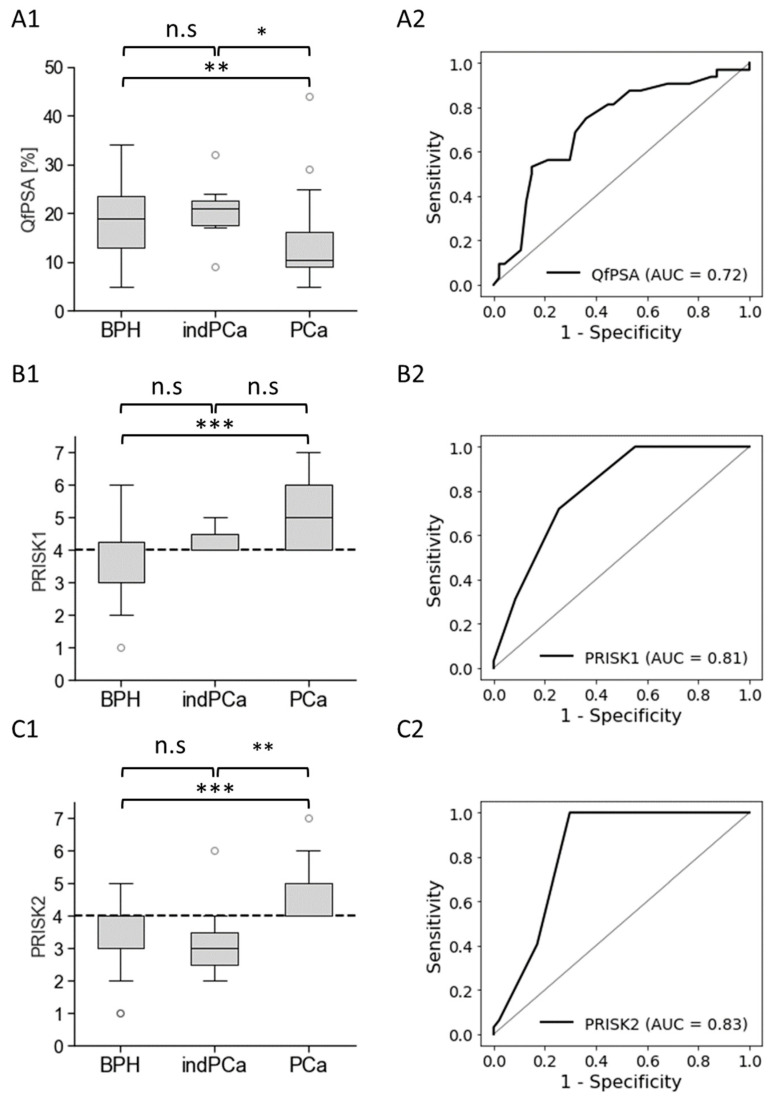
Developing protein- and cfDNA-based biomarker panels. Results of the classical protein-based PCa biomarker QfPSA (**A1**,**A2**) were compared to newly developed PCa risk scores 1 (PRISK1, **B1**,**B2**) and 2 (PRISK2 (**C1**,**C2**)). PCa risk scores considered assessments of QfPSA, cfDNA/mL, patient’s age, and methylated *RASSF1A*, *MIR129-2*, *NRIP3*, and *SOX8* DNA sequences/mL. Data are illustrated as box plots (**A1**–**C1**) for the BPH cohort (BPH, *n* = 40) compared to patients with indolent (indPCa, *n* = 7) and clinically significant PCa (PCa, *n* = 32) with tPSA values ranging from 2 to 15 ng/mL. Box plots consist of the median as “centre value”, the 25th and 75th percentiles as box edges, and the 10th and 90th percentiles as whisker boundaries. Data points outside whisker boundaries are shown as circles. The symbol * indicates significant differences with *p* < 0.05 (*), *p* < 0.01 (**), or *p* < 0.001 (***). The abbreviation n.s. (not significant) marks no significant differences between the analysed cohorts. (**B1**,**B2**,**C1**,**C2**) Cutoff values are displayed as dashed lines. (**C1**,**C2**) Cutoffs for PRISK1 were selected to detect all clinically significant and indolent PCa patients (100% overall SEN). The resulting SPE for BPH patients was 52.5%. (**C1**,**C2**) PRISK2 was developed to achieve maximum specificity of 70% for BPH patients and to decrease the number of detected indolent PCa patients with 100% SEN for clinically significant PCa patients. (**A2**–**C2**) ROC curve analyses were conducted, comparing BPH and indolent PCa cohorts with clinically significant PCa patients. The area under the curve (AUC) is indicated in parentheses.

Furthermore, we analysed the diagnostic performance of cfDNA-based biomarkers combined with the PSA-based biomarker QfPSA and developed two additional PCa risk scores, which included assessments of QfPSA, cfDNA/mL, patient’s age, and methylated *RASSF1A*, *MIR129-2*, *NRIP3*, and *SOX8* DNA sequences/mL. Score 1 (PRISK1) was created to detect all clinically significant and indolent PCa patients (100% SEN). We also generated a PCa risk score 2 (PRISK2) to further increase the SPE for BPH patients with 100% SEN for clinically significant (but not for indolent) PCa patients. Results were compared to the QfPSA analysis alone as shown in Figure 5.

**Figure 6:**
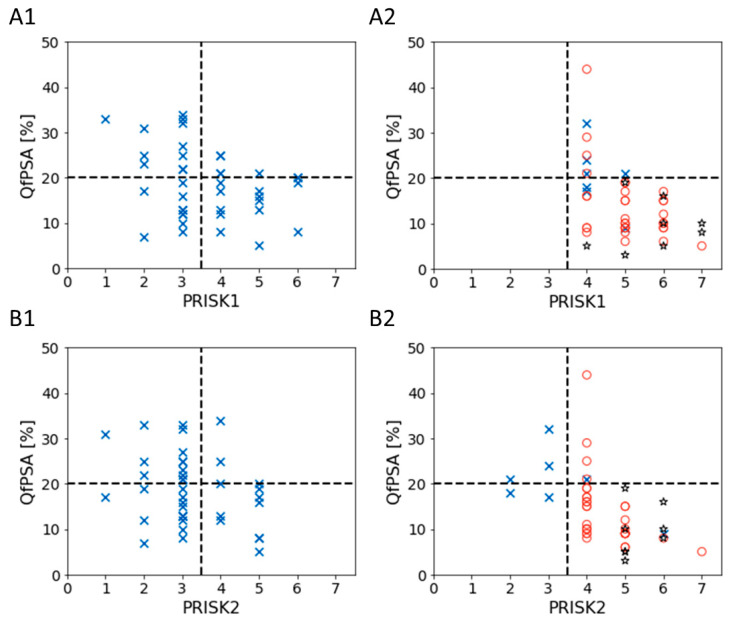
Comparisons of newly developed PCa risk scores (PRISK1 and PRISK2) with QfPSA alone. PRISK1 (**A1**,**A2**) and PRISK2 (**B1**,**B2**) were compared to QfPSA in 2D illustrations for BPH ((**A1**,**B1**), *n* = 40) and PCa patients (**A2**,**B2**). PCa patients were subdivided into indolent PCa (blue crosses, *n* = 7) and clinically significant PCa patients with tPSA values ranging from 2 to 15 ng/mL (red circles, *n* = 32) and tPSA values ≥ 15 ng/mL (black stars, *n* = 8). Cutoff values for QfPSA (>20%) and PRISK1 and 2 (<4) are shown as dashed lines. Biomarker panels consisted of QfPSA, cfDNA/mL, patient’s age, and methylated *RASSF1A*, *MIR129-2*, *NRIP3*, and *SOX8* DNA sequences/mL.

PRISK1 and PRISK2 were significantly increased for clinically significant PCa compared to BPH patients (*p* < 0.001) and, in the case of PRISK2, compared to indolent PCa patients (*p* < 0.01). The AUC values of PSA-dependent PRISK1 (0.81) and PRISK2 (0.83) were increased compared to QfPSA analysis alone and the corresponding PSA-independent scores piRISK1 and piRISK2 (Figure 5 and Figure S2). The newly developed PCa risk scores piRISK and PRISK demonstrated significantly improved performances compared to the PSA-based biomarkers tPSA and QfPSA, showing an increased diagnostic specificity and better indication for prostate biopsy, especially within the diagnostic grey area of tPSA in the range of 2 to 15 ng/mL (Figure 6 and Figure S3) [21,22,31].

For QfPSA alone, an SPE of 40% (16 out of 40 BPH patients negative) and a SEN of 87.5% (28 out of 32 PCa patients positive) were calculated (cutoff > 20%). Compared to QfPSA alone, SEN and SPE were increased in PSA-independent scores piRISK1 and piRISK2. Both scores had a SEN of 100% for clinically significant PCa patients (32 out of 32 PCa patients positive) and an SPE of 47.5% (19 out of 40 BPH patients) for piRISK1 (cutoff < 3) or 57.5% (23 out of 40 BPH) for piRISK2 (cutoff < 3). While piRISK1 was developed to detect all PCa patients including indolent PCa as positives, three out of seven (42.9%) indolent PCa patients were detected as negatives using piRISK2.

PSA-dependent scores PRISK1 and PRISK2 further increased SPE compared to QfPSA analysis alone and PSA-independent scores piRISK1 and piRISK2. An additional five BPH patients (52.5% SPE) and four clinically significant PCa patients (100% SEN) were correctly classified with PRISK1 (cutoff < 4), compared to QfPSA alone. Applying PRISK2, an additional 12 BPH patients (70% SPE) and 4 clinically significant PCa patients (100% SEN) were detected (cutoff < 4) compared to QfPSA alone. Moreover, five out of seven PCa patients with indolent disease (71.4%) were classified as negative using PRISK2, compared to four out of seven PCa patients (57.1%) identified using QfPSA alone. Alternatively, PRISK1 was developed to detect all PCa patients, including indolent PCa, as positive.

Using the newly developed OBBPA-ddPCR-based biomarker panel, eight plasma samples from patients with tPSA values ≥ 15 ng/mL and clinically significant PCa disease were analysed as positive controls. All eight samples were positive for QfPSA, piRISK1, piRISK2, PRISK1, and PRISK2 (Figure 6 and Figure S3).

Interestingly, at the time of blood sampling no PCa was detectable in six patients with tPSA values ranging from 2 to 15 ng/mL and two patients with tPSA values ≥ 15 ng/mL based on (for some patients multiple) preceding prostate biopsies. Despite this, prospectively determined piRISK1/2 and PRISK1/2 values were positive for all eight aforementioned patients and PCa was detected 4 to 19 months after initial blood sampling through additional prostate biopsies. For instance, one patient had a tPSA value of 7.0 ng/mL, QfPSA value of 15%, a normal digital rectal examination, and was initially diagnosed with atypical small acinar proliferation (ASAP). However, all newly developed PCa risk scores were above the respective cutoffs. For this patient, PCa (GS 7) was detected 9 months after the initial blood sampling by a repeated prostate biopsy followed by radical prostatectomy. A second patient of this group had a tPSA value of 11.5 ng/mL, QfPSA value of 15%, a normal digital rectal examination, and no malignancy was detectable during the initial prostate biopsy at the time of blood sampling. However, elevated piRISK and PRISK scores were estimated for this patient. Using magnetic resonance fusion prostate biopsy, PCa was diagnosed in this patient 19 months after the initial biopsy (GS 10). It is important to note that SEN and SPE calculations as well as ROC curve analyses of the present study were based on patient classification according to the latest histopathological information until data analyses of the present study were completed (September 2023).

Moreover, we analysed the patient cohort with unknown prostate cancer status (*n* = 45). Using PRISK1, 10 out of 45 (22.2%) patients were negative and, thus, prostate biopsies could be unnecessary. With PRISK2, 20 out of 45 (44.4%) prostate biopsies may be unnecessary without missing of any clinically significant PCa patients. In the case of QfPSA analysis alone, 19 out of 45 (42.2%) biopsies could be unnecessary, however, with the risk of missing clinically significant PCa patients. A longer follow-up period is required to verify the categorisation of these 45 patients based on the new PCa risk scores, since no histological findings are yet available.

## 4. Discussion

In the current study, we established a multiplexed OBBPA-ddPCR assay which is based on the assessment of five epigenetic biomarkers. The new approach aims to increase SEN and SPE of prostate cancer detection and optimise the indication for the following prostate biopsy. Primer pairs were designed to include two to three 5′-CpG sites and a 3′-end of a minimum of five nucleotides without any 5′-CpGs to ensure PCR biases above 90% together with sufficient amplification of unmethylated DNA sequences to counteract the susceptibility of methylation-specific amplification to false-positive results [32,33,34,35,36,37,38].

To evaluate the performance of the newly developed multiplexed OBBPA-ddPCR assay, a pilot study was conducted. We compared PSA-based scoring of BPH patients with indolent and clinically significant prostate cancer patients with tPSA values ranging from 2 to 15 ng/mL. Interestingly, there were no significant differences in tPSA levels between the studied patient groups, emphasising the common problem for urologists to decide whether or not to perform a prostate biopsy. Insufficient SPE leads to an increased number of unnecessary prostate biopsies which detect low-risk indolent prostate tumours [5,11]. Moreover, tPSA measurements are insufficient to detect all clinically significant prostate tumours [10]. The results of this study reflect the limitation of tPSA-based PCa diagnostics, since SPE of tPSA analysis was only 5% for the BPH cohort and 28.6% for indolent PCa patients, while SEN was 96.9% for the clinically significant PCa group (cutoff 4 ng/mL). Hence, there is an urgent need for additional biomarkers to improve SEN and SPE and provide more reliable indications for prostate biopsy.

We analysed blood-plasma-derived cfDNA methylation for five biomarker sequences: *RASSF1A*, *CCDC181*, *MIR129-2*, *NRIP3*, and *SOX8*. *RASSF1A* DNA promoter hypermethylation is detected in different types of malignancies and has been discussed as a potential diagnostic biomarker [39,40,41,42,43]. There is limited information about the significance of DNA hypermethylation of the remaining biomarkers. Recent studies showed that the differential expression and DNA methylation pattern of these genes may be used as diagnostic and prognostic biomarkers for different types of cancer, e.g., breast cancer (*NRIP3* [44], *MIR129-2* [45,46]), hepatocellular carcinoma (*SOX8* [47]), oesophageal cancer (*MIR129-2* [48]), lung cancer (*MIR129-2* [49]), and colorectal cancer (*MIR129-2* [50,51]). Interestingly, the *SOX* gene family is generally discussed as having oncogenic properties and SOX overexpression can be associated with poor prognosis [52]. However, results for SOX8 are scarce (in particular for PCa). *SOX8* is differentially expressed in primary gliomas, being upregulated in oligodendrogliomas and low-grade astrocytomas, but downregulated in glioblastomas, compared to healthy adult brain [53]. The *SOX8* promotor is hypermethylated in cervical cancer [54] and hepatocellular carcinoma [47]. Consequently, *SOX8*’s regulation and significance in cancer are very complex and should be explored in future investigations. In this study, although *CCDC181* failed to discriminate between BPH and PCa patients, the remaining biomarkers exhibited gradually increasing numbers of methylated DNA fragments/mL for healthy individuals, BPH patients, and PCa patients. According to initial in silico biomarker analyses, *CCDC181* DNA methylation differences were only observable in PCa tumour tissues compared to healthy prostate tissue but not within blood-derived cfDNA. This finding underlines the importance of cfDNA methylation analysis to be used in addition to the data from normal and malignant tissues for liquid biopsy DNA-methylation-based biomarker selection. Since no single epigenetic biomarker alone was able to sufficiently distinguish between BPH and PCa patients, we developed two PSA-independent PCa risk scores, piRISK1 and 2. The scores consist of known PCa risk factors (patient’s age [30] and amount of cfDNA [55]) as well as the newly developed DNA methylation biomarkers *RASSF1A*, *MIR129-2*, *NRIP3*, and *SOX8*. Furthermore, two PSA-dependent prostate cancer risk scores were developed (PRISK1 and PRISK2) which combine known PCa risk factors QfPSA [56], patient’s age [30], and the amount of plasma cfDNA [55] with methylated *RASSF1A*, *MIR129-2*, *NRIP3*, and *SOX8* cfDNA sequences/mL.

We note limitations of the present study. The follow-up of indolent PCa patients could only be conducted for a limited period of time and disease progression may occur in this group in the future. Hence, piRISK1 and PRISK1 were developed to identify all PCa patients with 100% SEN, regardless of the distinction between clinically significant and indolent cancers. For all patients examined in this study for whom histopathological data were already available, 47.5% of the conducted biopsies could have been prevented, according to the PSA-independent risk score piRISK1 without missing even one PCa case. Moreover, two additional BPH patients (52.5% SPE) were classified as true negative using PSA-dependent PRISK1. In conclusion, patients with negative piRISK1 or PRISK1 have a low risk of developing cancer and disease monitoring may be an appropriate strategy for these cases. To emphasise the difference, QfPSA-based diagnostics with a 20% cutoff may prevent 40% of unnecessary prostate biopsies (40% SPE), but three clinically significant PCa cases (identified as positive with our multiplexed OBBPA-ddPCR assay) would have been overlooked (87.5% SEN). These results are supported by the meta-analysis study, which demonstrated SEN of 70% and SPE of 55% for QfPSA, making it unsuitable for single-biomarker-based PCa detection [56].

Using PSA-independent piRISK2 or PSA-dependent PRISK2, unnecessary prostate biopsies could be further reduced by 17.5% or 30%, respectively, compared to QfPSA analysis alone, without overlooking even one clinically significant PCa patient. The scores were devised to identify only clinically significant, and not indolent, PCa. PRISK2 may further reduce unnecessary prostate biopsies, minimise overdiagnosis of indolent PCa, and simultaneously decrease the number of undetected clinically significant PCa diseases, compared to single-PSA-based analysis. A multicentre study by Catalona et al. (2011) investigated patients with tPSA values in the range of 2 to 10 ng/mL to evaluate the performance of the prostate health index (phi), a scoring system based on tPSA, fPSA, and [−2] proPSA, with SEN and SPE of 90% and 31%, respectively [57]. Considering only patients of the present study in the same tPSA range of 2 to 10 ng/mL, PRISK2 resulted in increased SEN and SPE of 100% (27 out of 27 clinically significant PCa positive) and 71.9% (23 out of 32 BPH negative), respectively. Although the number of examined samples was limited, the high diagnostic potential of the newly developed risk scores was demonstrated by the identification of eight patients who were diagnosed with clinically significant PCa up to 19 months after the initial blood sample analysis. This also shows the challenge of testing the positive predictive value of DNA methylation biomarkers when cancer can be discovered years later.

Altered methylation patterns are shown to occur very early during the onset of carcinogenesis, when a tumour can be missed even during a biopsy at this stage. Given that changes in DNA methylation occur at a very early stage of cancer, the absence of aberrant DNA methylation should imply a low tumour risk with high negative predictive value. In the present study, this means that 52.5% of the performed biopsies could have been avoided using PRISK1. Moreover, 70% of biopsies could be postponed using PRISK2, without overlooking a clinically significant PCa patient. However, further studies with an independent patient cohort are required to validate the diagnostic performance and negative predictive value of the investigated PCa risk scores over a prolonged period of several years. The OBBPA-ddPCR workflow, which requires time-consuming sample-processing steps (cfDNA isolation and bisulphite modification) and a two-stage detection process, needs to be simplified for clinical application. Further studies are planned to optimise the dPCR detection step by multiplexing and to evaluate the automatisation of the workflow by microfluidic chip integration.

## 5. Conclusions

In the present study, a multiplexed OBBPA-ddPCR assay was optimised and established, including cfDNA methylation analyses of *RASSF1A*, *MIR129-2*, *NRIP3*, and *SOX8* target sequences. A pilot study analysis of blood plasma samples from 90 healthy individuals (controls), 40 BPH patients, and 39 PCa patients revealed a gradual increase in cfDNA methylation for control, BPH, and PCa cohorts using biomarkers *RASSF1A*, *MIR129-2*, *NRIP3*, and *SOX8*. Classical PCa risk factors (QfPSA and patient’s age) were combined with cfDNA-based parameters (cfDNA amount/mL and methylated *RASSF1A*, *MIR129-2*, *NRIP3*, and *SOX8* copies/mL) to develop PCa risk scores PRISK1 and 2 with improved SEN and SPE. Both PCa risk scores were developed to further reduce unnecessary prostate biopsies and PCa overdiagnosis. PRISK1 exhibited an increased SEN (100% for indolent (*n* = 7) and clinically significant PCa (*n* = 32)) and SPE (52.5% for BPH patients) compared to single-marker analyses of tPSA and QfPSA. Using PRISK2, 70% of unnecessary prostate biopsies (28 out of 40 BPH patients) may be prevented without missing any clinically significant PCa patients. Notably, no prostate biopsy could be prevented within the BPH group when cutoffs of single-marker analyses of tPSA and QfPSA were selected to detect all clinically significant PCa patients. Our findings demonstrate synergistic effects of classical protein-based and cfDNA-based biomarkers, provide improved SEN and SPE, and provide reliable reasons to proceed to prostate biopsy in patients within the diagnostic grey area of tPSA measurements.

## Data Availability

Data supporting reported results can be found online (Supplementary Tables S1–S3).

## Supplementary Materials

The following supporting information can be downloaded at: https://www.mdpi.com/article/10.3390/cancers16071324/s1, Table S1: Characteristics of analysed patient samples; Table S2: Primer and probe sequences; Table S3: Prostate cancer risk scores and corresponding cutoffs; Figure S1: Multiplex PCR bias optimisation: adjustments of MgCl_2_ concentration and annealing temperature; Figure S2: PSA-independent biomarker panels; Figure S3: Comparisons of newly developed PSA-independent PCa risk scores (piRISK1 and piRISK2) with QfPSA alone.

## Abbreviations

ASAP	atypical small acinar proliferation
AUC	area under the curve
BPH	benign prostatic hyperplasia
BRCA1 and 2	breast cancer gene 1 and 2
CCDC181	coiled-coil domain containing 181
cell-free DNA	cfDNA
CPD	copy per droplet
ddPCR	digital droplet PCR
FAM	carboxyfluorescein
fPSA	free (unbound) prostate-specific antigen
GS	Gleason score
HEX	hexachloro-fluorescein
MIR129-2	microRNA 129-2 gene
NRIP3	nuclear receptor interacting protein 3 gene
OBBPA-ddPCR	optimised bias-based preamplification–digital droplet PCR
PCa	prostate carcinoma
phi	prostate health index
piRISK1 and 2	PSA-independent prostate carcinoma risk scores 1 and 2
PRISK1 and 2	PSA-dependent prostate carcinoma risk scores 1 and 2
QfPSA	percentage of free PSA compared to total PSA
RASSF1A	Ras association domain family member 1 gene
ROC curve analyses	receiver operating characteristic curve analyses
SEN	diagnostic sensitivity
SOX8	SRY-box transcription factor 8 gene
SPE	diagnostic specificity
TCGA	The Cancer Genome Atlas
tPSA	total serum prostate-specific antigen
