# Supplementary material for: Blood-Based DNA Methylation Analysis by Multiplexed OBBPA-ddPCR to Verify Indications for Prostate Biopsies in Suspected Prostate Cancer Patients
Source: Cancers (Basel). 2024 Mar 28;16(7):1324. doi: 10.3390/cancers16071324 (PMC11010987; doi:10.3390/cancers16071324)
Supplement: Supplementary file 1 [file cancers-16-01324-s001.zip › Supplementary Figures.pdf]

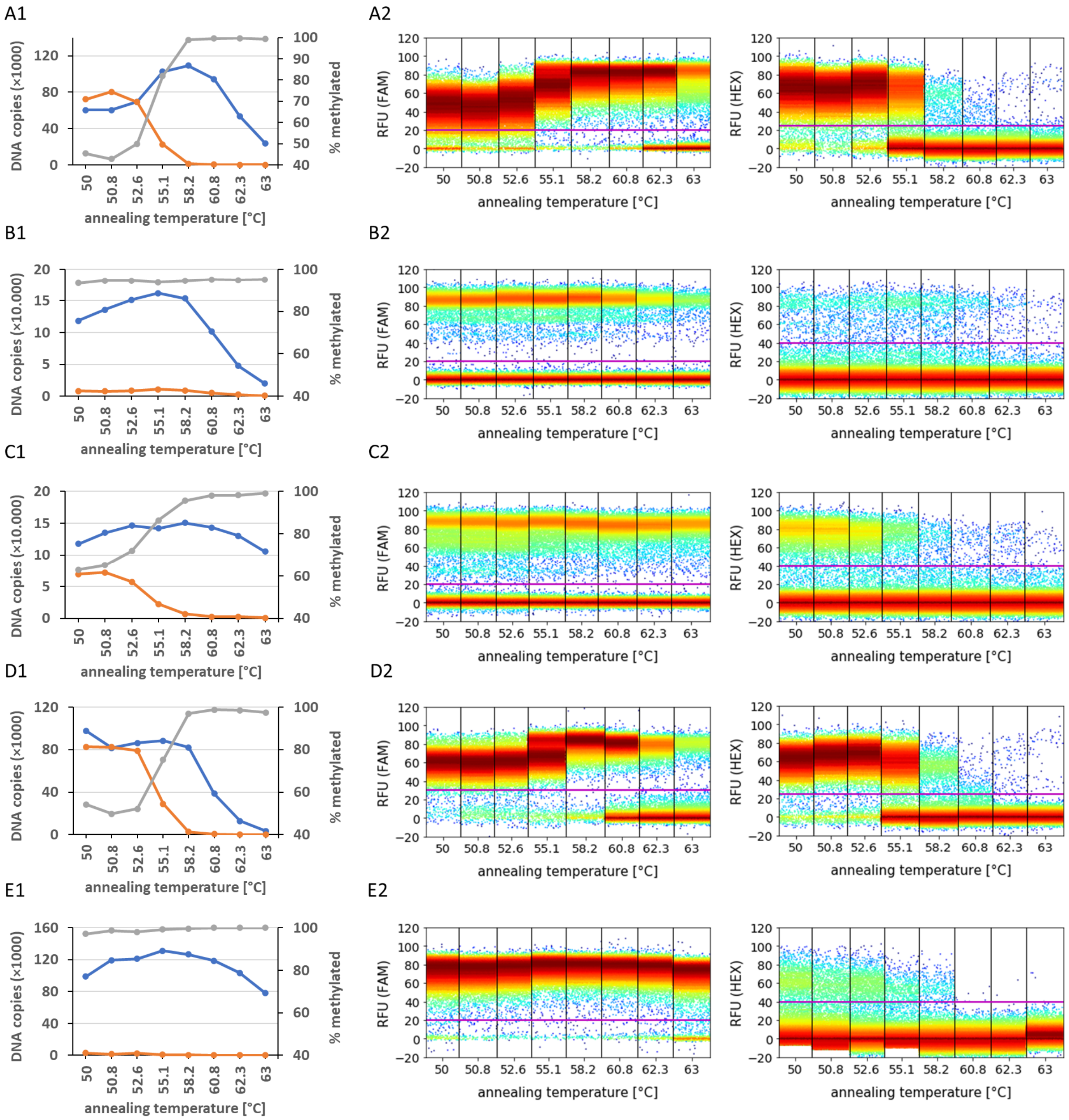

Figure S1. Multiplex PCR bias optimization: adjustments of MgCl2 concentration and annealing temperature. The impact of varying primer annealing temperatures on the amplification rate of methylated and unmethylated *RASSF1A* (A1,A2), *CCDC181* (B1,B2), *MIR-129-2* (C1,C2), *NRIP3* (D1,D2), and *SOX8* (E1,E2) DNA sequences is shown for a Mg2+ concentration of 3.5 mM. (A1–F1) DNA fragment numbers of methylated (blue) and unmethylated DNA (orange) as well as the percentage of methylated DNA relative to the total DNA after 12 cycles of preamplification (PCR bias, grey) are displayed as function of varying primer annealing temperatures. (B1,B2) Fluorescence signals of FAM fluorophores for methylated (left) and HEX fluorophores for unmethylated DNA sequences (right) are shown depending on the primer annealing temperature.

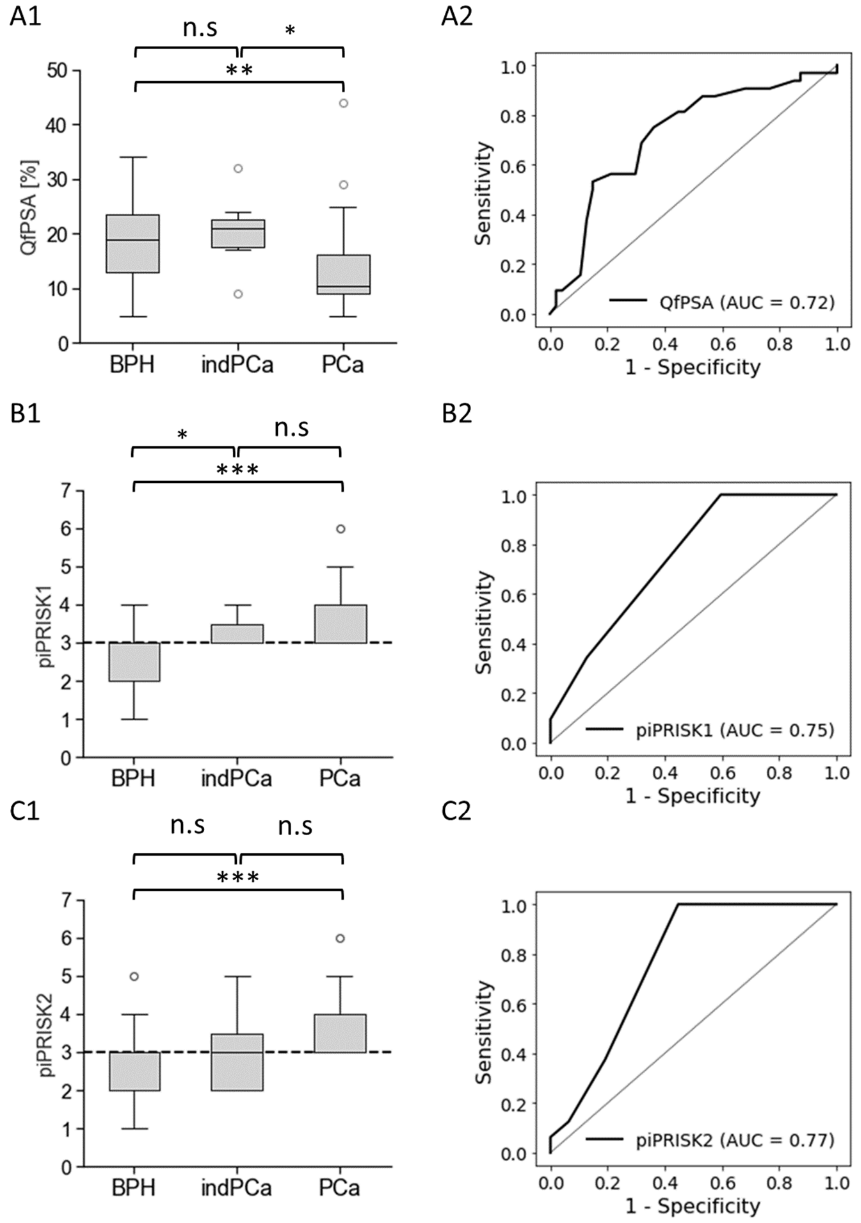

Figure S2. PSA-independent biomarker panels. Results of the classical protein-based PCa biomarker QfPSA (A1,A2) were compared to the newly developed PSA-independent PCa risk scores 1 (piRISK1, (B1,B2)) and 2 (piRISK2, (C1,C2)). PCa risk scores consisted of cfDNA/mL, patient's age, and methylated *RASSF1A*, *MIR129-2*, *NRIP3*, and *SOX8* DNA sequences/mL. Data is illustrated as boxplots (A1–C1) for the BPH cohort (BPH, n = 40) compared to patients with indolent (indPCa, n = 7) and clinically significant PCa (PCa, n = 32). Box plots consist of the median as 'center value', the 25th and 75th percentiles as box edges, and the 10th and 90th percentiles as whisker boundaries. The symbol * indicates significant differences with $p < 0.05$ (*), $p < 0.01$ (**), or $p < 0.001$ (***). The abbreviation n.s. (not significant) marks no significant differences between the analysed cohorts. (B1,C1) Cutoff values are displayed as dashed lines. (B1,B2) Cutoffs for piRISK1 were selected to detect all clinically significant and indolent PCa patients (100% overall SEN). The resulting SPE for BPH patients was 47.5%. (C1,C2) piRISK2 was developed to achieve maximum specificity of 57.5% for BPH patients and to decrease the number of detected indolent PCa patients with 100% SEN for clinically significant PCa patients. (A2–C2) ROC curve analyses were conducted, comparing BPH and indolent PCa cohorts with clinically significant PCa patients. The area under the curve (AUC) values are indicated in parentheses.

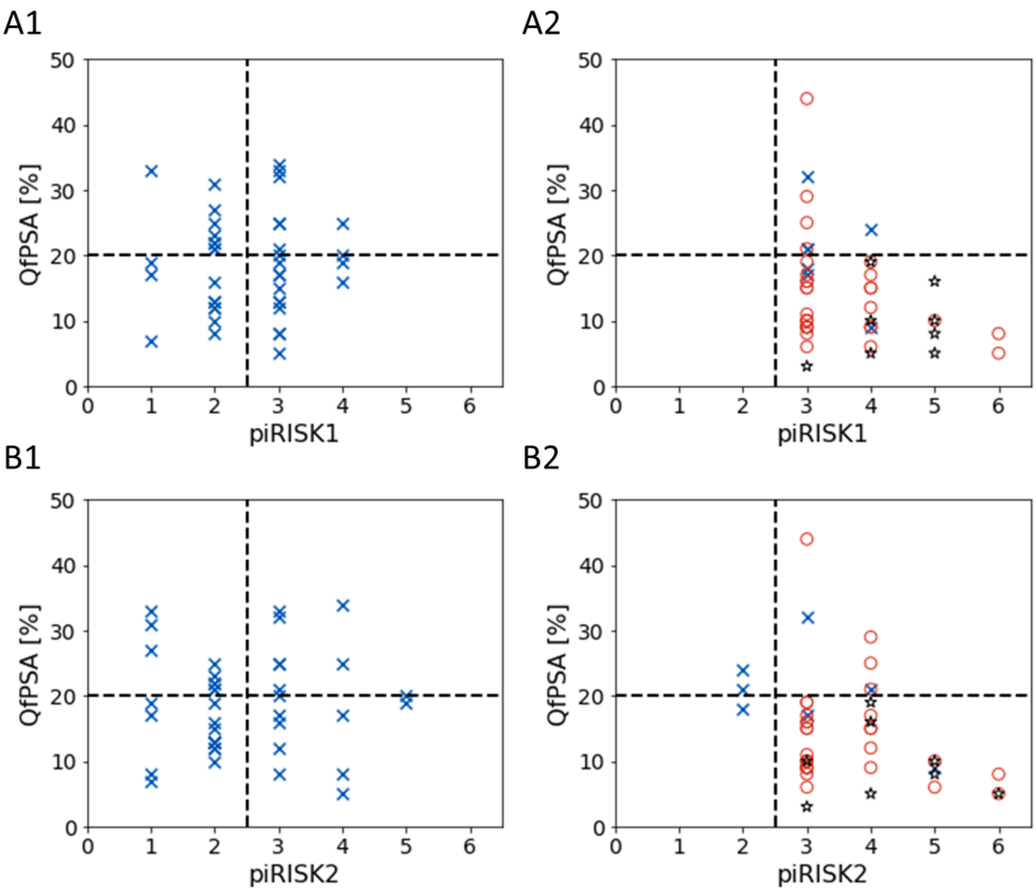

Figure S3. Comparisons of newly developed PSA-independent PCa risk scores (piRISK1 and piRISK2) with QfPSA alone. The PSA-independent scores piRISK1 (A1,A2) and piRISK2 (B1,B2) were compared with QfPSA in 2D illustrations for BPH ((A1,B1), n = 40) and PCa patients (A2,B2). (A2,B2) PCa patients were subdivided into indolent PCa (blue cross, n = 7) and clinically significant PCa patients with tPSA values ranging from 2 to 15 ng/mL (red circles, n = 32) and tPSA values ≥15 ng/mL (black stars, n = 8). Cutoff values for QfPSA (>20%) and piRISK1 and 2 (<3) are shown as dashed lines. Biomarker panels consisted of cfDNA/mL, patient's age, and methylated *RASSF1A*, *MIR129-2*, *NRIP3*, and *SOX8* DNA sequences/mL.
